# Draft Genome Sequence of *Lactobacillus delbrueckii* subsp*. bulgaricus* LBB.B5

**DOI:** 10.1128/genomeA.01090-16

**Published:** 2016-10-06

**Authors:** Zoltan Urshev, Karima Hajo, Leonardo Lenoci, Peter A. Bron, Annereinou Dijkstra, Wynand Alkema, Michiel Wels, Roland J. Siezen, Svetlana Minkova, Sacha A. F. T. van Hijum

**Affiliations:** a LB Bulgaricum Plc., R & D Center, Sofia, Bulgaria; b Center for Molecular and Biomolecular Informatics, Radboudumc, Nijmegen, the Netherlands; c NIZO food research, Ede, the Netherlands; d Microbial Bioinformatics, Ede, the Netherlands

## Abstract

*Lactobacillus delbrueckii* subsp*. bulgaricus* LBB.B5 originates from homemade Bulgarian yogurt and was selected for its ability to form a strong association with *Streptococcus thermophilus*. The genome sequence will facilitate elucidating the genetic background behind the contribution of LBB.B5 to the taste and aroma of yogurt and its exceptional protocooperation with *S. thermophilus*.

## GENOME ANNOUNCEMENT

*Lactobacillus delbrueckii* subsp*. bulgaricus* is invariably found in yogurt produced in the Balkan region in combination with *Streptococcus thermophilus* (1) and occasionally it has been also isolated from raw milk and plant material (2). *L. delbrueckii* subsp*. bulgaricus* strain LBB.B5 was originally isolated in 1969 in the village of Dalboki (Bulgaria, GPS coordinates 42.481258, 25.770522) from homemade yogurt and maintained in the culture collection of LB Bulgaricum PLC (Sofia, Bulgaria). The strain has been used in industry in combination with *S. thermophilus* to produce Bulgarian yogurt with its typical taste and aroma (3, 4). Strain LBB.B5 was chosen for industrial application for yogurt starters for its ability to form a strong association with selected *S. thermophilus* strains (5). Only very few other pairs of *L. delbrueckii* subsp*. bulgaricus* and *S. thermophilus* strains cultures proved to be able to form stable associations, pointing to the rareness of this phenomenon (5). Such continuously co-cultured starters were even assigned the term “symbiotic.” Complete understanding of the nature of protocooperation between *L. delbrueckii* subsp*. bulgaricus* and *S. thermophilus* needs underpinning from genomic data. Also, there are very few genome-based analyses that point to the properties that make a given *L. delbrueckii* subsp*. bulgaricus* strain suitable for industrial application (6).

The genome sequence of strain LBB.B5 was determined using a 101-bp paired-end library with Illumina Hiseq 2000 technology (Illumina, San Diego, CA) at BaseClear (Leiden, the Netherlands). A total of 2,545,204 reads were generated and assembled by IDBA-UD 1.1.1 (7) into 129 contigs of which 101 contigs were larger than 500 bp. Functional annotations of the predicted genes were performed using the RAST annotation server (8). Sequencing of strain LBB.B5 led to a final draft genome sequence of 1,777,882 bp, which contains 1,841 open reading frames (ORFs). Five rRNA genes and 74 tRNA genes were identified next to 33 pseudogenes. The G+C content of the genome is 49.8%. Based on its genome sequence, strain LBB.B5 was predicted to synthesize serine and cysteine, two of the four important amino acid synthesis pathways essential to the industrial application of a strain (9). Strain LBB.B5 was found to contain a complete set of genes for phosphate transport/homeostasis, peptide transport, and *de novo* fatty acids synthesis.

The genome information of *L. delbrueckii* subsp*. bulgaricus* LBB.B5 presented here will be useful for further studies into the genomic determinants for protocooperation and fermentation performance.

### Accession number(s).

The sequence data for the genome described here have been deposited at GenBank under the accession number LUGK00000000 and RefSeq accession number NZ_LUGK00000000.

## References

[1] Urshev Z, Michaylova M, Minkova S, Isawa K 2002 High genetic diversity of *Lactobacillus bulgaricus* and *Streptococcus thermophilus* in home-made yogurt samples, Poster A50. *In* Seventh Symposium on Lactic Acid Bacteria, Egmond aan Zee, the Netherlands, September1 to 5. doi:10.13140/2.1.1389.3760.

[2] Michaylova M, Minkova S, Kimura K, Sasaki T, Isawa K 2007 Isolation and characterization of *Lactobacillus delbrueckii* ssp. *bulgaricus* and *Streptococcus thermophilus* from plants in Bulgaria. FEMS Microbiol Lett 269:160–169. doi:10.1111/j.1574-6968.2007.00631.x. 17257163

[3] Kondratenko M, Goranova L, Kondareva S, Gjoscheva B 1976 Zahl, Art und Menge der freien Aminosäuren in bulgarischer Sauermilch und deren Säureweckern. Detsche Molkerei Zeitung 22:649–653.

[4] Gyosheva B 1982 Compounds forming the aroma complex in Bulgarian sour milk. Milchwissenschaft 37:267–269.

[5] Kondratenko M, Kondareva S, Gyosheva B, Vlaykovska A, Shishkova I, Toteva N, Goranova L 1979 Method for obtaining combination starters for Bulgarian yoghurt. U.S. patent 4,156,019.

[6] Hao P, Zheng H, Yu Y, Ding G, Gu W, Chen S, Yu Z, Ren S, Oda M, Konno T, Wang S, Li X, Ji ZS, Zhao G 2011 Complete sequencing and pan-genomic analysis of *Lactobacillus delbrueckii* subsp. *bulgaricus* reveal its genetic basis for industrial yogurt production. PLoS One 6:e15964. doi:10.1371/journal.pone.0015964. 21264216 PMC3022021

[7] Peng Y, Leung HC, Yiu SM, Chin FY 2012 IDBA-UD: a *de novo* assembler for single-cell and metagenomic sequencing data with highly uneven depth. Bioinformatics 28:1420–1428. doi:10.1093/bioinformatics/bts174. 22495754

[8] Overbeek R, Olson R, Pusch GD, Olsen GJ, Davis JJ, Disz T, Edwards RA, Gerdes S, Parrello B, Shukla M, Vonstein V, Wattam AR, Xia F, Stevens R 2014 The SEED and the Rapid Annotation of microbial genomes using Subsystems Technology (RAST). Nucleic Acids Res 42:D206–D214. doi:10.1093/nar/gkt1226. 24293654 PMC3965101

[9] Liu E, Hao P, Konno T, Yu Y, Oda M, Zheng H, Ji ZS 2012 Amino acid biosynthesis and proteolysis in *Lactobacillus bulgaricus* reviewed: A genomic comparison. Comput Mol Biosci 2:61–77.

